# Uncommon Morphologic Types of Endometrial Cancer and Their Mimickers: How Much Does Molecular Classification Improve the Practice for Challenging Cases?

**DOI:** 10.3390/life14030387

**Published:** 2024-03-14

**Authors:** Ozlen Saglam

**Affiliations:** Department of Pathology, Oregon Health and Science University, 3181 SW Sam Jackson Park Rd., Portland, OR 97239, USA; osaglam@hotmail.com

**Keywords:** spindle cells, clear cells, papillary, biphasic, microglandular morphology

## Abstract

The previous endometrial cancer (EC) FIGO staging primarily relied on the extent of the disease spread into the anatomical sites at diagnosis. The most recent one (2023) incorporates clinicopathological features such as histological subtype, tumor grade, the extent of lymphovascular space invasion (LVI), and, when available, molecular subtypes of EC. The emphasis on accurate histological typing, tumor grading, and the molecular features of the cancer is stronger than ever. This review addresses challenging diagnostic patterns in the histologic subtyping and grading EC under five categories: 1. EC with spindle cells, 2. EC with clear cells, 3. EC with a papillary architecture, 4. EC with a biphasic morphology, and 5. EC with a microglandular architecture. The morphological features differentiating low- and high-grade cancers are discussed, along with relevant clinical work-ups. Recent molecular genetic findings regarding the diagnosis and prognosis of the disease and the results of related clinical trials are summarized. The potential challenges in the evaluation of LVI follow these sections. The final section of the review includes an overview of the literature on incorporating molecular subtypes of EC into clinical practice.

## 1. Introduction

Endometrial cancer (EC) is the sixth most common cancer among women worldwide, with 417,000 new cases and 97,000 deaths in 2020 [1]. In the United States, uterine cancer incidence and mortality rates are still increasing in contrast to other cancer types [2]. The three most prevalent cancers among females in 2022 were breast cancer, uterine corpus cancer (891,560), and thyroid cancer. The racial disparity is significant in uterine cancer treatment [3]. The 5-year relative survival rate of 81% across all stages is higher for white women at 84% compared to black women at 63%. The latter group is more likely to have an aggressive tumor type [4] and be diagnosed at advanced disease stages [5]. EC is assigned a FIGO grade based on the degree of glandular differentiation if endometrioid or mucinous differentiation is present. Grade 1 tumors exhibit ≤5% solid nonglandular, nonsquamous growth; grade 2 tumors range from 6% to 50%; and grade 3 tumors are >50%. FIGO grading is not used for all other EC types, which carry an intrinsic tumor grade. By definition, serous, clear-cell, and undifferentiated carcinomas and carcinosarcomas are high-grade cancers (aggressive type) [6]. The lack of improvement in survival, especially for aggressive histological subtypes, has led to more research on the molecular genetics of EC in the last two decades.

The molecular classification of EC by the Cancer Genome Atlas (TCGA) Research Network includes four categories: *POLE* ultramutated, microsatellite instability (MSI) hypermutated, low-copy number, and high-copy number cancers [7]. Applying these findings to clinical cases [8] and risk assessments by integrating molecular and clinicopathological parameters [9] have paved the road for the FIGO 2023 staging update [10]. The current FIGO staging includes not only anatomic sites of disease spread but also a histological subtype, tumor grade, and the quantity of lymphovascular space involvement (LVI). When molecular results are available, FIGO 2023 incorporates them in the staging of EC. The staging procedure has become more personalized, with an intensified role of pathology for directing further disease management. In this review, the unusual patterns of low-grade EC and uncommon aggressive cancer types are presented (Table 1) in comparison with the more frequent diagnostic mimickers sharing mutual architectural patterns. The differential diagnosis includes the morphological features crucial for accurate diagnosis. The molecular genetic findings of these entities from the recent literature are covered after histopathologic findings. A summary of the diagnostic challenges in the pathological quantification of LVI and the literature on how to incorporate EC molecular subtypes into clinical practice follows these sections.

## 2. Endometrial Cancer with Spindle Cells

This group includes low-grade (FIGO grades 1 and 2) and high-grade (aggressive) carcinomas. Among the low-grade cancers, endometrioid adenocarcinoma with spindle cells (ECSC) and corded and hyalinized endometrioid adenocarcinoma (CHEC) are rare forms of EC that can be challenging to diagnose, especially in small biopsies.

### 2.1. Endometrioid Carcinoma with Spindle Cells (ECSC)

**Figure 1:**
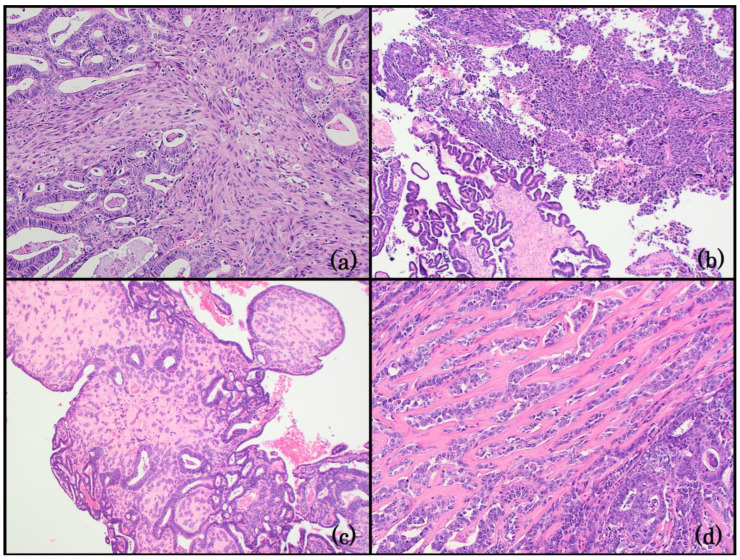
Group 1: EC with spindle cells. (**a**) Endometrioid carcinoma with spindle cells. The spindle cells merge with the glandular component throughout the lesion. No significant cytological atypia is present in either spindle cells or glandular epithelium (200x); (**b**) the sarcomatous and carcinomatous elements of carcinosarcoma are spatially distinct and have high-grade morphology (200x); (**c**) CHEC: the neoplastic cells are embedded in a hyalinized stroma, forming cords and trabeculae (40x); (**d**) the nuclear features are similar in CHEC and conventional adenocarcinoma component (right lower quadrant) (200x). The spindle cell component is not in the figure.

Diagnosed in the endometrium and ovaries, ECSC can have a prominent spindle component in some samples, making up more than 90% of the lesion [11]. Spindle cells in diffuse patterns are not spatially distinct and merge with the glandular component throughout the lesion (Figure 1a).

Focal nuclear palisading, necrosis, or keratinization within the spindle cell areas are additional morphological features. The spindle and epithelial cells have low-grade nuclei. A low mitotic activity can be present in the spindle cell component. The histological grading depends on the grade of the adenocarcinoma component (FIGO grading) per expert consensus [11]. No established histologic grading system exists for ECSC. Cytokeratin can be focal- or diffuse-positive in the spindle cell component of ECSC, as p16 is patchy-positive. p53 has a normal staining pattern.

Low-grade Endometrial Stromal Sarcoma (LGESS) with glandular differentiation and carcinosarcoma are in the differential diagnosis of ECSC. LGESS with glandular differentiation usually has a focal epithelioid component. The neoplastic cells resemble the stromal cells of a normal proliferative-type endometrium, with a delicate vascular pattern like spiral arterioles of the endometrium. The tumor cells can arrange themselves in concentric whorls around vessels. Thick bands of collagen and hyalinization are components of the neoplasm [12]. A diffuse CD10 positivity supports the LGESS diagnosis. Carcinosarcoma is usually diagnosed in postmenopausal women (mean age: 70 years), especially those with an extrauterine disease [13]. In contrast to ECSC, carcinosarcoma with a spindle cell component has high-grade and spatially distinct epithelial and mesenchymal components (Figure 1b). P53 expression by immunohistochemistry is a mutant type in both elements.

### 2.2. Corded and Hyalinized Endometrioid Carcinoma (CHEC)

CHEC contains epithelioid cells in cords or clusters embedded in a hyalinized stroma [14] (Figure 1c,d). An osseous (24%) or chondroid metaplasia (4.5%) and a myxoid matrix (38.5%) can be a component of the lesion [15,16]. The glandular, corded, and spindled cells merge at some foci. Low-grade endometrioid carcinoma with squamous differentiation is usually associated with CHEC, and the histological grade depends on the grade of conventional endometrioid carcinoma. Cytokeratin and EMA are often weakly to moderately positive in the corded and spindled components [17]. Beta-catenin nuclear expression in all components and the loss of E-cadherin expression in the spindle and corded epithelioid cells [18] suggest the epithelial–mesenchymal transition. p53 has a normal/wild-type expression pattern in most cases (89%) [19]. CHEC is usually a low-grade carcinoma (FIGO grades 1 and 2), yet high-grade CHEC made up 12.5% of the samples in a small case series [16]. *CTNNB1* (beta-catenin) mutations involving exon 3 are common in low-grade and high-grade CHEC [18,20]. The majority of cases fall into no specific molecular profile of TCGA classification.

The differential diagnoses of CHEC include carcinosarcoma, LGESS with sex cord-like differentiation, and uterine tumor resembling ovarian sex cord tumor (UTROSCT). LGESS with sex cord-like differentiation has tumor cells focally arranging themselves in cords, nests, and trabeculae that resemble sex cord-like elements. *PHF1* genetic rearrangements can be present in some cases [21]. UTROSCT is a well-circumscribed nodular lesion with morphological patterns resembling ovarian sex cord tumors. The growth patterns and cytological features are variable, including sheets, cords, nests, trabeculae, and tubules without an endometrial stromal component [22]. A smooth muscle component can be present within the lesion or at the periphery by the entrapment of the surrounding myometrium. The neoplastic cells have scant-to-abundant pale, eosinophilic, or foamy cytoplasm. Nuclei are usually mildly atypical and sometimes pushed eccentrically, gaining rhabdoid cell morphology. Multiple-lineage positivity, including sex cord, epithelial, smooth muscle markers, CD10, ER, and PR, is possible in varying percentages [23]. A panel approach is necessary in the application of ancillary studies.

## 3. EC with Clear Cells

All histological subtypes of EC can have focal cytoplasmic clearing, especially after neoadjuvant chemotherapy for advanced-stage cancers. Endometrioid cancers with focal mucinous or clear changes, clear-cell carcinoma (CCC), and yolk sac tumor (YST) in postmenopausal women are primary uterine neoplasms in this category.

### 3.1. Endometrioid Cancer with Focal Mucinous Change

**Figure 2:**
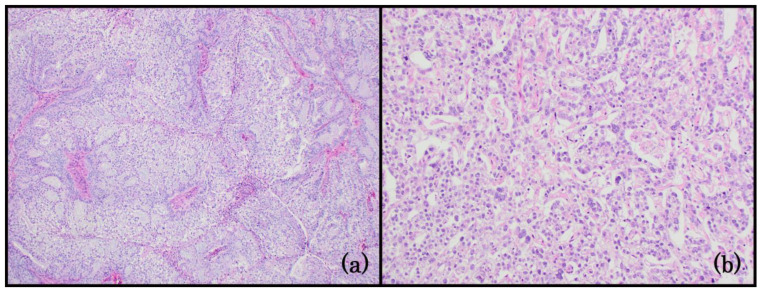
Group 2: EC with clear cells. (**a**) Endometrioid adenocarcinoma with mucinous features. Intracytoplasmic and extracellular mucin are prominent in this field. The neoplastic cells have cytoplasmic clearing and well-defined cell borders at the center of the image. Low-grade nuclear features are noticeable throughout the lesion (100x); (**b**) the glandular and solid patterns of an endometrial yolk sac tumor. The neoplastic cells have significant nuclear atypia and cytoplasmic clearing (100x).

This subtype is composed of varying amounts of mucin-secreting glandular epithelium (Figure 2a) and includes at least focal endometrioid-type adenocarcinomas or atypical endometrial hyperplasia (Endometrial Intraepithelial Neoplasia—EIN). Scattered goblet cells are frequently present. Nuclei are usually of a low grade in all components. Mucinous differentiation in endometrioid-type adenocarcinoma is more common in older patients and is associated with an increased likelihood of myometrial invasion. This finding does not affect the disease stage or risk of recurrence [24]. By immunohistochemistry, CK7 is diffusely positive, and CK20, CEA, and CDX2 can be focal-positive. The differential diagnosis is metastatic adenocarcinoma from the gastrointestinal tract, especially in cases with minimal endometrioid carcinoma components. The immunohistochemical profile overlaps with metastatic upper or lower gastrointestinal tract tumors. Conventional endometrioid adenocarcinoma or EIN can be focal, and adequate sampling is essential to rule out metastatic disease from the gastrointestinal tract and primary endometrial gastric/gastrointestinal-type adenocarcinoma [25]. The latter cancer is a rare and relatively new diagnostic entity listed in the latest edition of the WHO Blue Book for Female Genital Tumors. The morphological features resemble the endocervical counterpart, containing pyloric-type mucinous epithelium and goblet cells. Immunohistochemically, CK7, CEA, MUC6, PAX 8, CK20 (50%), CDX2 (50%), and estrogen receptor (25%) are positive, and Napsin A is negative in a small case series. The mutation-type p53 staining is present in 50% of cases [25]. Limited clinical experience suggests aggressive behavior. Human Papilloma Virus (HPV)-dependent endocervical adenocarcinoma extending into the uterine cavity can also mimic endometrioid adenocarcinoma with mucinous differentiation. The presence of endocervical adenocarcinoma in situ is supportive of primary endocervical adenocarcinoma. P16 is diffusely positive in HPV-dependent endocervical cancers and patchy positive in endometrioid cancer. In situ hybridization test for high-risk HPV is helpful in the diagnosis of primary endocervical adenocarcinoma in samples with equivocal p16 results [26].

### 3.2. Endometrioid Carcinoma with Clear Cells

The clear cells in low-grade endometrioid carcinoma are associated with the presence of squamous cells, intracytoplasmic glycogen, neoadjuvant therapy, or NOS. The nuclei are uniformly low grade, and secretary-type changes may be present as sub-or supranuclear vacuoles. The immunophenotype is the same as the conventional low-grade endometrioid carcinoma, with patchy p16 positivity and wild-type p53 expression. CCC is in the differential diagnosis of all cancers with clear cells. CCC has tubulocystic, papillary, and solid architectural patterns, usually in combination. A significant cytological atypia, at least focally, should be present. The cell types include low cuboidal, polyhedral, hobnail, signet ring-like, mucinous, or flattened cells. Napsin A, HNF-1ß, and AMACR are positive in varying percentages [27]. ER and PR are either negative or focal weak positive. The advanced-stage EC with clear cells after neoadjuvant therapy can be challenging to diagnose. Clinical history, pretreatment biopsies, and immunohistochemistry aid in correct histologic typing in small samples.

### 3.3. Somatically Derived YST

Endometrial YST has similar histological patterns to its ovarian counterpart, including microcystic/reticular, glandular (Figure 2b), solid, papillary, and hepatoid types. An admixture of histologic patterns is frequently observed. Around 45% of uterine YSTs are associated with somatic components (epithelial carcinoma), and 15% are associated with a second germ cell component. Schiller–Duval bodies can be present in 23% of samples [28]. A marked cytological atypia and easily identifiable mitotic figures are typical findings. The cytoplasm might be scant to abundant and either clear or eosinophilic. Subnuclear or supranuclear cytoplasmic vacuolation can be prominent or a focal feature. A polyphenotypic marker expression (epithelial and germ cell markers) is frequent. Alpha-fetoprotein (AFP) is a specific marker of YST. EMA can be positive or negative [29]. GATA3 is only expressed in primitive patterns of YST (reticular/microcystic, polyvesicular, and polyembryoma) but not in differentiated forms (glandular and hepatoid) [30]. The benefit of germ cell-appropriate chemotherapy in somatically derived YST is not definite for postmenopausal women.

## 4. EC with a Papillary Architecture

Low-grade endometrioid carcinoma with a papillary architecture, serous carcinoma (SC), and mesonephric-like carcinoma (MLC) are ECs with either predominantly papillary architecture or in combination with other architectural patterns.

### 4.1. Low-Grade Endometrioid Carcinoma with a Predominant Papillary Architecture

**Figure 3:**
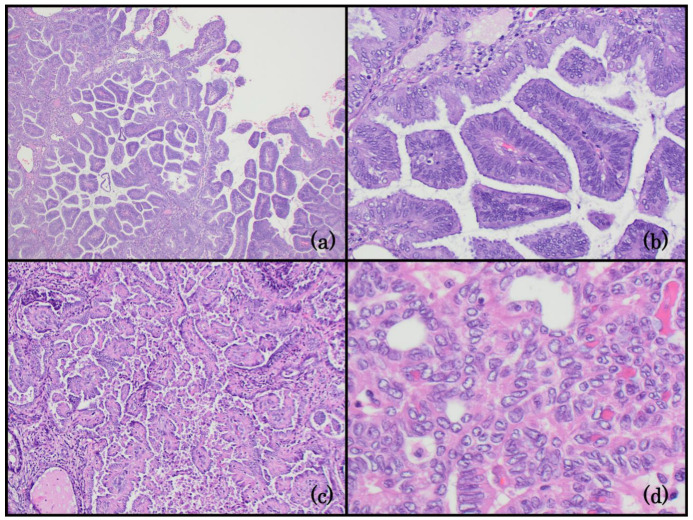
Group 3: EC with a papillary architecture. (**a**) Endometrioid carcinoma with a papillary architectural pattern is the superficial component of the lesion (100x); (**b**) the nuclei in the papillary endometrioid carcinoma are uniform, columnar, and perpendicular to the basement membrane (400x); (**c**) mesonephric-like carcinoma of the endometrium with a papillary architecture. The variable-sized papillae have delicate fibrovascular cores (200x); (**d**) the nuclei of the mesonephric-like carcinoma are crowded and overlapping. They have open chromatin and nuclear grooves, resembling papillary thyroid carcinoma (400x).

Endometrioid carcinoma with a predominant papillary architectural pattern (Figure 3a) has a scant glandular component. The nuclei are stratified, columnar, and vertically oriented to the basement membrane (Figure 3b). Nucleoli are inconspicuous. Focal mucinous metaplasia is a frequent finding. The histological grade is FIGO grade 1 or 2 [31]. A well-differentiated endometrioid adenocarcinoma with long, slender, and finger-like papillae and no associated budding cells is called endometrioid adenocarcinoma with a villoglandular pattern. In mixed cases with a typical endometrioid appearance, the villoglandular component usually constitutes the superficial component. Tumors with villoglandular patterns in the myoinvasive front are associated with a higher frequency of LVI and lymph node metastasis in some series [32]. The immunostaining profile of low-grade endometrioid carcinoma with a papillary architecture is identical to that of typical low-grade endometrioid carcinoma, with a wild-type p53 staining pattern, diffuse ER and PR expression, and patchy p16 positivity. Endometrial SC is a high-grade cancer with marked nuclear pleomorphism, round nuclei with prominent eosinophilic nucleoli, numerous mitotic figures, and conspicuous apoptosis. Immunohistochemically, P53 has mutation pattern staining, and p16 is diffusely positive [33]. In TCGA molecular classification, SC is within the high-copy number group and characterized by copy number amplifications of *PIK3CA*, *ERBB2* [34], and *TP53* mutations (90%) [35].

### 4.2. Mesonephric-like Carcinoma (MLC)

MLCs make up to 3% of all ECs [36]. Multiple architectural patterns, including tubular, glandular, retiform, papillary (Figure 3c), solid, ductal, glomeruloid, and spindle cell-type patterns, can be present in a lesion with various combinations [37]. Dense eosinophilic secretions typically fill the lumens of glands or tubules. The nuclei are often crowded, open, and vesicular and resemble those of papillary thyroid carcinoma with pseudoinclusions and grooves (Figure 3d). The mitotic activity is variable. Mucinous or squamous differentiation is typically not present. No histologic grading system exists, but MLC is in the aggressive histologic group of the 2023 FIGO staging update. The localization of the lesion is either in the endometrium or on the uterine wall. Multiple immunostains, including GATA3, PAX8, CD10 (luminal), TTF1, calretinin, and AR, are expressed [38], and ER, Napsin A, and AMACR are negative in MLC. P53 has a wild-type staining pattern. Somatic *KRAS* mutations, *BRAF* hotspot mutations, *PIK3CA*, and *PTEN* mutations are frequent [39]. Small case series support its aggressive behavior, and clinical experience with this type is limited. Diagnosis usually takes place at an advanced disease stage. Recurrences can occur at local or distant sites with a predilection of pulmonary metastasis [40]. The differential diagnoses of MLC are endometrioid adenocarcinoma with papillary architecture, CCC, and cervical mesonephric carcinoma (CMC) extending into the uterine corpus. The nuclei in low-grade endometrioid adenocarcinoma are pseudostratified and columnar-type nuclei. The presence of squamous or mucinous differentiation supports endometrioid adenocarcinoma diagnosis. CCC has tubulocystic and solid architectural patterns in addition to papillary architecture. The papillae have a delicate and sometimes hyalinized core. The morphologic and immunostaining profiles of MLC and CMC overlap significantly. CMC can be associated with a background of mesonephric remnants or hyperplasia. In primary uterine cases, the bulk of the tumor is within the uterine cavity. TTF1 expression is more frequent in MLC than in CMC [40].

## 5. EC with a Biphasic Morphology

This rare EC has both low- and high-grade carcinoma components. Mixed neuroendocrine carcinoma (NEC) with low-grade endometrioid cancer and dedifferentiated EC have high- and low-grade carcinoma components.

### 5.1. Mixed NEC and Endometrioid Carcinoma

**Figure 4:**
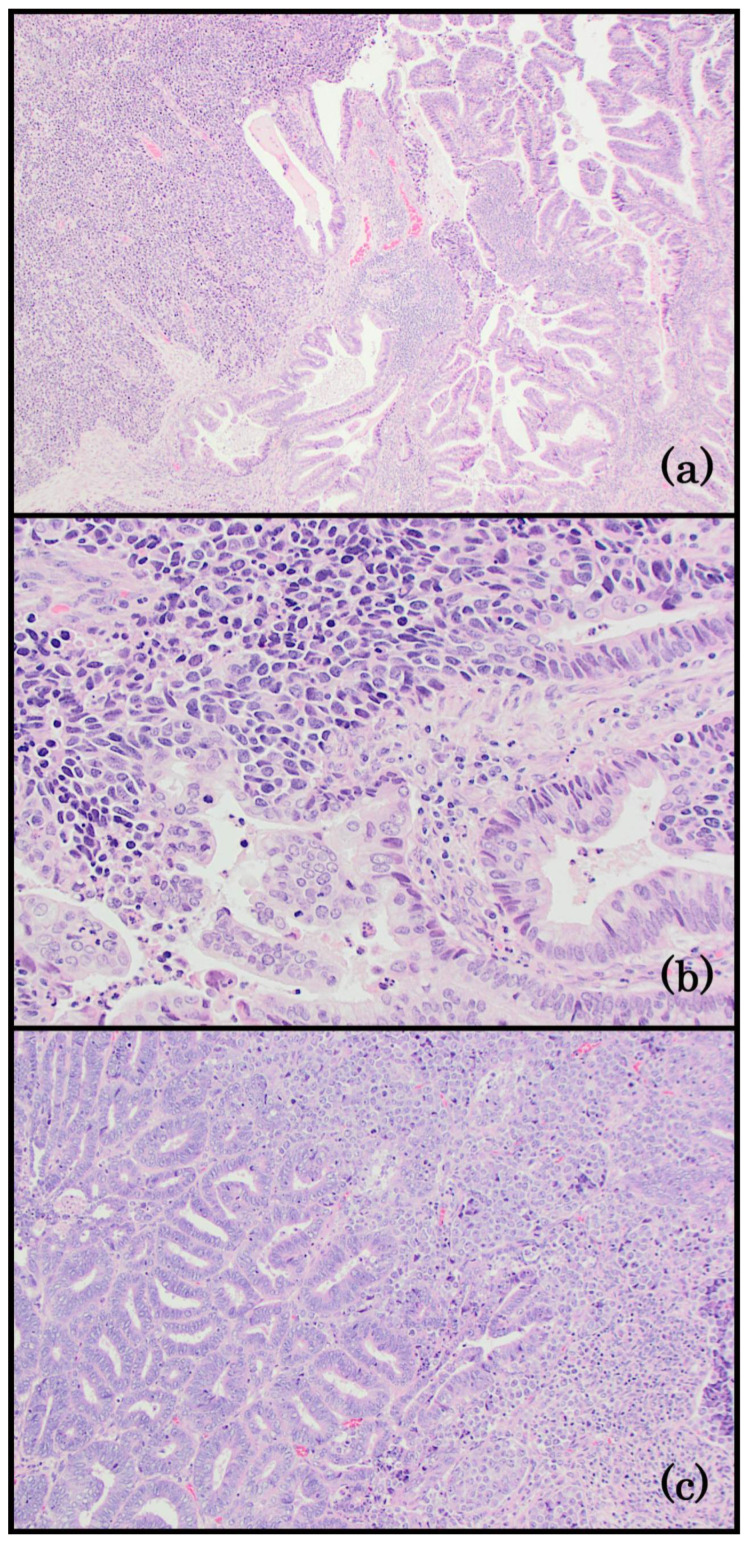
Group 4: EC with a biphasic morphology. (**a**) Mixed neuroendocrine carcinoma (left) and endometrioid carcinoma (right). A sharp demarcation separates the two components (100x); (**b**) the small-cell neuroendocrine carcinoma (left) has nuclear molding, hyperchromasia, and a high nuclear/cytoplasmic ratio. Apoptosis is a prominent feature. The glandular component (right) merges with the neuroendocrine cells in this field (400x); (**c**) dedifferentiated endometrial carcinoma. The differentiated component is a FIGO grade 1 endometrioid carcinoma (left). The undifferentiated neoplastic cells (right) are small, uniform, and dyscohesive (200x).

The morphological features of endometrial NEC are not different from those of NECs at other anatomical sites. The majority of endometrial NECs are mixed with other carcinoma subtypes, more frequently with an endometrioid adenocarcinoma (Figure 4a,b) [41,42]. The pathology report should include individual tumor types and their approximate percentages. Small-cell NECs contain neoplastic cells with a high nuclear-to-cytoplasmic ratio, nuclear hyperchromasia, molding, and crush artifacts. There might be focal spindling of the nuclei. Large-cell NECs have appreciable cytoplasm and coarse nuclear chromatin, often with prominent nucleoli. The architectural patterns include insular/nested, trabecular, pseudoglandular, and solid types. Broad zones of necrosis and high mitotic activity are frequent findings in small- and large-cell NECs. One or multiple neuroendocrine markers (synaptophysin, chromogranin, CD56, and INSM1) are typically positive in the NEC component and negative in low-grade endometrioid carcinoma. NEC of the endometrium is rarely TTF1-positive (1 sample out of 18 samples) and associated with microsatellite instability (8 samples out of 18) [43]. NEC of the endometrium is molecularly heterogeneous and can fall into all four subgroups of TCGA molecular classification [44]. The differential diagnoses are FIGO grade 3 endometrioid carcinoma and dedifferentiated/undifferentiated carcinoma. High-grade endometrioid carcinoma has at least focal gland/acini formation. The presence of squamous and mucinous differentiation in these areas supports the diagnosis of endometrioid carcinoma. The identification of the NEC component is prognostically significant. The 5-year overall survival is 68.8% for FIGO grade 3 endometrioid carcinoma and 38% for mixed NEC and endometrioid carcinoma [45]. Patients with pure NEC have a worse prognosis than patients with mixed cell tumors [46].

### 5.2. Dedifferentiated Carcinomas

Dedifferentiated carcinomas have differentiated (FIGO grade 1 or 2) and undifferentiated components. The differentiated component is usually more superficial and sharply demarcated from the undifferentiated carcinoma (Figure 4c). Undifferentiated cancers have monomorphic small- to intermediate-sized dyscohesive cells arranged in sheets without apparent glandular elements. A component of FIGO grade 1 or 2 endometrioid carcinoma (differentiated cancer) is present in 40% of undifferentiated carcinomas [47]. There might be focal-marked nuclear pleomorphisms, broad zones of necrosis, rhabdoid cell morphology, multinucleation, spindling, and abrupt keratinization within the undifferentiated component. Focal or diffuse EMA and cytokeratin expression are present in 80% of samples. The neuroendocrine marker expression should be limited to less than 10% of the lesion by definition. A lack of PAX8 expression (83%) [48] and abnormal mismatch repair (MMR) protein results are frequent in undifferentiated carcinoma samples (69%) [49]. By immunohistochemistry, a variable loss of BRG-1 (*SMARCA4*), INI-1 (*SMARCB1*), and *ARID1A* can be supplemental to the diagnosis [50]. The 5-year overall survival for dedifferentiated/undifferentiated carcinoma is worse than FIGO grade 3 endometrioid adenocarcinoma, with 84% for stage I/II disease, 38% for stage III disease, and 12% for stage IV disease [49]. Lymphoma and plasmacytoma are differential diagnoses if the differentiated component is not in the sample. A hematopoietic marker expression of CD45 (lymphoma) or CD138 (plasmacytoma) supports the diagnosis of hematologic malignancies. The morphology of *SMARCA4*-deficient uterine sarcoma can be indistinguishable from dedifferentiated carcinoma [51]. However, patients are from younger age groups (median: 24 years). The presence of a focal and vague phyllodiform architecture and stromal hyalinization favors sarcoma diagnosis. Microsatellite instability reportedly has not been detected in *SMARCA4*-deficient sarcoma [51].

## 6. EC with a Microglandular Pattern

**Figure 5:**
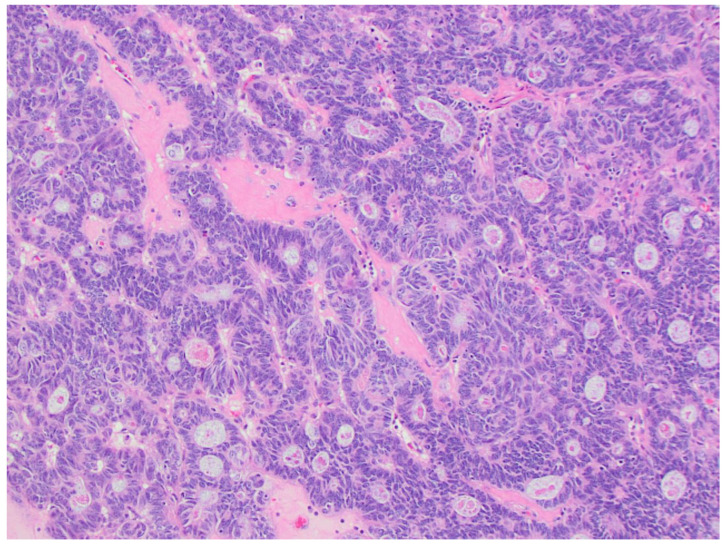
Group 5: Endometrioid adenocarcinoma with microglandular pattern contains mucin-producing columnar cells, forming small acini (100x).

In small biopsies, it can be a diagnostic challenge to differentiate this variant of EC from endocervical microglandular hyperplasia (EMH). Endometrioid carcinoma with a microglandular pattern is a disease that predominantly affects postmenopausal women [31]. EMH is associated with oral contraceptive usage in younger patients. Endometrioid carcinoma with a microglandular pattern contains small- to medium-sized, back-to-back glands lined by one or more layers of columnar or cuboidal cells (Figure 5). Intraluminal mucin and squamous differentiation are frequent findings. Endometrioid carcinoma with a microglandular pattern can present in pure form or mixed with typical EC located on the surface of the lesion [52]. Immunohistochemistry has a limited role in distinguishing endometrioid carcinoma with a microglandular pattern from EMH due to the overlapping expression patterns of CEA, p63, p16, and vimentin [53]. A negative PAX2 result supports the diagnosis of EC. The presence of residual endometrial glands or stroma in fragments containing the tumor, the lack of reserve cells, or poorly formed and variably distributed intracytoplasmic vacuoles are the morphological features that lead to a definitive EC diagnosis.

## 7. Lymphovascular Invasion

Substantial LVI (≥5 vessels involved) is one of the pathological parameters in the 2023 FIGO staging of EC that upstages the disease. PORTEC-1 and PORTEC-2 clinical trials have concluded that EC with substantial LVI is associated with a significantly higher risk of recurrence and shorter overall survival [54,55]. The 5-year risk of pelvic lymph node recurrence is 15.3% in cases with substantial LVI compared with 1.7% and 2.5% for no LVI and focal LVI, respectively. Per the current Collage of American Pathologist’s guidelines, the count of vessels with LVI is on the single slide with the highest number of vessel involvement and not the sum of involved vessels on all slides. Preanalytic factors such as laparoscopic hysterectomy samples and tissue contamination during the grossing procedure may complicate the evaluation of LVI [56]. In these samples, artificially displaced tumor fragments within the vascular spaces are large, unattached to the vessel wall, without admixed fibrin, and lack the inflammatory response seen around vessels. An abundant stroma in the tumor within a vessel also strongly favors contamination. In samples with true LVI, the neoplastic cells should be present not only in large vessels but also in small capillaries. The risk of artificial displacement is higher in polypoid tumors and less likely in flat lesions.

**Figure 6:**
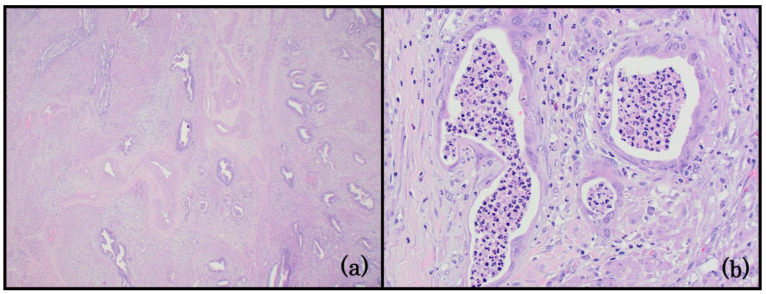
(**a**) The Microcytic Elongated and Fragmented (MELF) Pattern of Myometrial Invasion (40x); (**b**) a fibromyxoid stroma and inflammatory cells surround elongated glands with outpouchings in the MELF pattern of myometrial invasion. The lining epithelium is partly flattened (200x).

In myoinvasive EC, a prominent fibromyxoid stromal reaction can accompany outpouchings, elongation, and fragmentation of neoplastic glands in some samples [57]. This pattern of myometrial invasion is called the Microcytic Elongated and Fragmented (MELF) pattern (Figure 6a,b). The lining epithelium is either flattened or can have eosinophilic cytoplasm. In a low-power examination, neoplastic glands with a flattened lining epithelium might be misinterpreted as LVI. The MELF pattern of myometrial invasion is associated with a larger tumor size, a myometrial invasion of more than 50% of the uterine wall, a high number of LVI, lymph node metastasis, a papillary architecture, mucinous differentiation, and a higher risk of isolated tumor cells in sentinel lymph nodes of FIGO grade 1 EC [58,59]. PORTEC-1 and PORTEC-2 clinical trials have failed to show the independent impact of the MELF pattern on the risk of recurrence in early-stage EC [60].

## 8. Integration of Molecular Classification of EC into Clinical Management

The treatment and prognosis of EC depend on the assessment of recurrence risk determined by applying clinicopathological parameters [61]. Among these parameters, morphologic subtyping and histological grading of EC, especially for high-grade cancers, have low reproducibility, even at expert levels [47,62]. In the past two decades, the clinical validation of molecular subtypes of EC has yielded a stepwise algorithm, the Proactive Molecular Risk Classifier for Endometrial Cancer (ProMisE) [63], which requires the testing of pathologic mutations of the endonuclease domain of polymerase epsilon (POLEmut), as well as the evaluation of p53 expression patterns and mismatched repair (MMR) protein levels by immunohistochemistry. The ProMisE algorithm divides EC into four molecular subtypes: 1. POLEmut EC, 2. mismatch repair-deficient (MMRd) EC, 3. no specific molecular profile (NSMP), and 4. P53 abnormal (p53abn) EC. *POLE* and MMR proteins have a role in the genetic stability of DNA replication by proofreading [64] and mismatch correction [65], respectively. Eleven discrete pathogenic variants of POLEmut have been identified so far, and two of which—P286R and V411L—are present in approximately two-thirds of POLEmut ECs. Five “hotspots” cover the vast majority (around 80%) of pathologic POLEmut ECs [66]. POLE and MMR defects result in high mutation rates with additional secondary abnormalities. Tumors with pathogenic POLEmut co-existent with MSI-High subtypes show genomic alterations characteristic of POLE-ultramutated ECs. In a pooled analysis, 13 ECs with DNA MMRd/MSI-High and POLEmut have a comparable 5-year recurrence-free survival to previously reported POLE-ultramutated ECs [66]. In another study, the 5-year recurrence-free survival of patients with MMRd-p53abn and POLEmut-p53abn stage I ECs is 92.2% and 94.1%, respectively. The results are significantly better from single-classifier p53abn EC and support the classification of MMRd-p53abn EC as MMRd and POLEmut-p53abn EC as POLEmut [67].

Around 7–12% of ECs demonstrate mutations in the exonuclease domain of POLE [7]. Patients with POLEmut EC are relatively young, have a low body mass index, and have the highest prevalence of FIGO stage I tumors [68]. The tumor morphology is of a high grade with scattered giant tumor cells, prominent lymphocytic infiltrates, and frequent LVI [69,70]. The histological subtypes of 43 *POLE*-mutated ECs include 79% endometrioid, 14% mixed-type, 5% serous, and 2% clear-cell carcinomas [71]. The majority of POLEmut endometrioid carcinomas are considered high-grade ECs (63%). However, FIGO grade 3 endometrioid carcinoma is a heterogenous disease and can fall into all four molecular subtypes (12.9% POLEmut, 20.7% p53abn, 30.2% NSMP, and 36.2% MMRd) [72]. The PORTEC-4a clinical trial includes patients with high–intermediate risk EC, with treatment recommendations based on an integrated molecular risk profile [73]. It will show whether omitting treatment in POLEmut EC is safe and cost-effective. The MMRd group makes up 25–30% of EC. MMR deficiency is significantly more frequent in high-grade endometrioid carcinomas than in low-grade endometrioid carcinomas (39.7% versus 24.7%) [74]. Almost half (44%) of undifferentiated carcinomas [75] and 16 to 66% of mixed ECs with an endometrioid carcinoma component have abnormal MMR protein expression [76].

Somatic MMR deficiency occurs sporadically in the majority of MMRd EC samples due to the hypermethylation of the *MLH1* promoter region and consequent epigenetic silencing. Only 10% of MMR deficiency is inherited as part of Lynch Syndrome. EC is often the sentinel cancer in a pedigree and provides an opportunity to detect syndromic disease [77]. Diagnosing the hypermutated MMRd/MSI category of EC can have important management implications. The MMR status influenced the response to the fertility-sparing treatment of EC by progesterone in some recent studies. Patients with MMRd tumors had lower complete or partial response rates than patients with EC expressing wild-type p53 in 6 months [78]. Resistance to conservative treatment is more frequent in MMRd than in MMR-proficient cases, without statistical significance [79]. The MMRd group also shows a high prevalence of deep myometrial invasion and LVI. The latter finding can justify sentinel lymph node sampling in this molecular group. The overall prognosis of MMRd ECs is intermediate. Patients with MMRd tumors respond to radiotherapy and receive significant survival benefits from chemotherapy. Predictive testing for MMRd tumors of all anatomical sites confirms eligibility for targeted treatment with immune checkpoint inhibitors [80].

Most p53abn ECs are serous-type and high-grade ECs, but this type also includes other histologic types and lower-grade tumors. P53-mutant ECs are often morphologically ambiguous, accounting for almost all serous ECs, most carcinosarcomas and mixed ECs, and half of all clear-cell ECs [81]. The clinical outcome is the poorest for this molecular subtype. Although, p53abn EC only accounts for 15% of all EC cases, it is responsible for 50–70% of EC mortality [82]. p53 immunohistochemistry reliably identifies samples with *TP53* mutation in ovarian and endometrial cancers [83,84]. The most common abnormal p53 pattern is overexpression (68%), followed by null type (9%) and cytoplasmic (2%) patterns [85]. Regardless of histological subtype, HER2 amplification has been identified as a therapeutic target in a subset of p53abn carcinomas [86]. The NSMP group does not have high mutational load or significant copy number variations and makes up the majority of ECs (40%), with a prototype of low-grade endometrioid carcinoma [81]. The risk of recurrence is not well established for this category. The subclassification of NSMP cases is currently under evaluation in the PORTEC-4a study [73].

## 9. Conclusions

The TCGA’s molecular classification of EC has provided a new approach to EC treatment. Clinical trials, including morphologic and molecular subtypes of EC, may tailor treatment modalities to avoid over- or undertreatment in subgroups of patients with EC. 

Our current knowledge about molecular features of uncommon subtypes is limited. The rarity of challenging morphologic subtypes prohibits the possibility of conducting large cohort studies in a single institution. Therefore, multicenter and prospective studies are imperative to elucidate the molecular features of uncommon subtypes and enhance our knowledge of these tumors. Accurate histological typing and tumor grading stand as pivotal clinicopathologic parameters in EC treatment, guiding the direction of molecular testing in future investigations. 

## Figures and Tables

**Table 1:** Uncommon subtypes of endometrial carcinoma and their differential diagnosis. AFP: alpha-fetoprotein. CCC: clear-cell carcinoma. GAS: gastrointestinal-type carcinoma. HPV: Human Papilloma Virus. LGESS: Low-grade Endometrial Stromal Sarcoma. MC: mesonephric carcinoma. MLC: mesonephric-like Carcinoma. UTROSCT: uterine tumor resembling ovarian sex cord tumor.

Uncommon Histologic Type	Mimicker	Diagnostic Work-Up	Molecular Features
Endometrioid Carcinoma with Spindle Cells	- Carcinosarcoma - LGESS with Glandular Differentiation	P53 P16 ER and PR	N/A
Corded and Hyalinized Endometrioid Carcinoma	- Carcinosarcoma - LGESS with sex cord differentiation - UTROSCT	Beta-catenin E-cadherin P53	*CTNNB1* Mutation
Endometrioid Carcinoma with Mucinous Differentiation	- Primary endometrial GAS and metastatic GAS - HPV-dependent endocervical adenocarcinoma	Overlapping immunophenotype with metastatic cancers CK7, CK20, CDX-2, p16	N/A
Endometrioid Carcinoma with Clear Cells	- CCC - Yolk sac tumor	Napsin A HNF-1ß AMACR	N/A
Endometrioid Carcinoma with Papillary Architecture	- Serous carcinoma - MLC	P16 ER and PR p53	N/A
Somatically Derived Yolk Sac Tumor	- CCC - Endometrioid carcinoma with clear cells	AFP Glypican SALL4 EMA	Shared mutations with epithelial component (limited data)
Mesonephric-Like Carcinoma	- Endometrioid carcinoma - CCC - Cervical MC	GATA3 CD10 TTF-1 Calretinin	*KRAS*, *BRAF PIK3CA*, and *PTEN* mutations
Mixed Endometrioid and Neuroendocrine Carcinoma	- FIGO grade 3 Endometrioid carcinoma - Dedifferentiated carcinoma	Synaptophysin Chromogranin CD56 INSM1	Heterogeneous, fall into all 4 molecular groups
Undifferentiated/Dedifferentiated Carcinoma	- FIGO grade 3 endometrioid carcinoma - Lymphoma - Plasmacytoma - *SMARCA4*-deficient uterine sarcoma	EMA BRG-1 INI-1 MMR proteins	*SMARCA4*, *SMARCB1*, *ARID1A* inactivating mutations
Endometrioid Carcinoma with Microglandular Pattern	Microglandular hyperplasia (small biopsies)	PAX2	N/A
